# An Unusual Case of Unexplained Infertility: Co-colonization of the Uterus and Seminal Fluid

**DOI:** 10.7759/cureus.101251

**Published:** 2026-01-10

**Authors:** Kami Mukenschnabl, Olivia Humpel, Tori E Abdalla, Ellen Wood

**Affiliations:** 1 Obstetrics and Gynecology, Mount Sinai Medical Center, Miami Beach, USA; 2 Medicine, Lake Erie College of Osteopathic Medicine, Bradenton, USA; 3 Internal Medicine, Temple University Hospital, Philadelphia, USA; 4 Reproductive Endocrinology and Infertility, IVFMD/South Florida Institute for Reproductive Medicine, Cooper City, USA

**Keywords:** bacteriospermia, chronic endometritis, endometrial biopsy, endometrial microbiome, in vitro fertilization, unexplained infertility

## Abstract

Chronic endometritis (CE) is defined as a persistent, mild inflammation of the endometrium induced by intrauterine bacterial infection. CE has been associated with infertility in patients with recurrent in vitro fertilization (IVF) failure. We report an unusual case of bacterial co-colonization of the endometrium and seminal fluid in a couple with unexplained infertility.

A 35-year-old woman presented to the office for infertility evaluation after 16 months of inability to conceive naturally using ovulation kits. Initial workup revealed adequate ovarian reserve with an anti-Müllerian hormone (AMH) level of 4.8 ng/mL, tubal patency on hysterosalpingogram, and normal semen analysis. The patient and her partner failed to conceive following three cycles of ovulation induction with intrauterine insemination (IUI). In preparation for IVF, an endometrial biopsy (EMB) was performed, and five CD138+ plasma cells per 10 high-power fields suggested CE. The patient underwent antibiotic therapy, yet EMB remained positive. At this time, the partner's semen culture was positive for *Enterococcus faecalis* and *Escherichia coli*. Endometrial microbiome metagenomic analysis (EMMA) and analysis of infectious chronic endometritis (ALICE) demonstrated co-colonization with the same bacteria seen on her partner's semen culture. Both the patient and her partner required multiple rounds of antibiotic therapy before successful conception via IVF.

This case demonstrates an unusual occurrence of bacterial co-colonization of the endometrium and seminal fluid in a couple with unexplained infertility, suggesting a potential pathway for CE development from bacteriospermia. The patient's EMMA/ALICE tests and the partner's semen cultures revealed the presence of the same bacteria. While current literature does not identify the development of CE from the bacteria in a partner's semen, there is an association between bacteria in semen and infertility.

In couples with unexplained infertility, thorough evaluation for CE with EMB and EMMA/ALICE can be performed in conjunction with a semen culture on the partner to explore potential co-colonization and guide dual-partner treatment.

## Introduction

Chronic endometritis (CE) is defined as a persistent, mild inflammation of the endometrium, characterized by edema, plasma cell infiltrates, and increased stromal cell density [1]. CE is often asymptomatic and is typically discovered incidentally during other diagnostic procedures, such as hysteroscopy or endometrial biopsy (EMB). When symptoms are present, they are non-specific and include pelvic pain, dysfunctional uterine bleeding, dyspareunia, and leukorrhea [2]. Due to the nature of these symptoms, or lack thereof, patients often go undiagnosed. 

Several structural factors are believed to contribute to the development of CE, including endometrial polyps, uterine leiomyomas, and endometrial hyperplasia [3]. The nature of CE continues to be studied; however, it is becoming widely accepted that it is an infectious process caused by bacteria and microbial dysbiosis within the uterine cavity, inducing chronic inflammation, and most cases are well-treated with antibiotics [3-5]. The bacteria most commonly linked to infectious CE include *Enterococcus*, *Staphylococcus*, *Streptococcus*, *Mycoplasma*, and *Ureaplasma* [2]. Risk factors that increase the likelihood of bacterial contamination of the uterus and the development of CE are intrauterine device (IUD) use and endometriosis [3,5].

EMB is the most reliable and definitive diagnostic method for CE. Diagnosis is confirmed when the EMB reveals five or more plasma stromal cells, stained with CD138, in 10 high-power fields (HPF). The presence of fewer than five plasma stromal cells in 10 HPF may suggest the presence of CE, but it is not diagnostic [6]. As CE is thought to be an infectious process caused by bacteria, if the EMB results are positive for CE, an endometrial microbiome metagenomic analysis (EMMA) and analysis of infectious chronic endometritis (ALICE) may be performed to identify the specific bacteria that are responsible for the condition and can guide targeted antibiotic therapy. The EMMA/ALICE testing can detect the presence of *Chlamydia trachomatis*, *Enterobacteriaceae*, *Enterococcus faecalis*, *Escherichia coli*, *Klebsiella pneumoniae*, *Mycoplasma genitalium*, *Mycoplasma hominis*, *Neisseria gonorrhoeae*, *Staphylococcus aureus*, *Streptococcus agalactiae*, *Streptococcus viridans*, and *Ureaplasma urealyticum* [7]. 

Although definitive diagnosis of CE is with an EMB, hysteroscopy is commonly used as a visual diagnostic tool for CE. Typical findings on hysteroscopy in a patient with CE include endometrial micropolyposis and strawberry spots [8]. Micropolyposis, which is observed in up to 67% of women with infertility and/or recurrent pregnancy loss, is thought to be associated with CE. Strawberry spots, characterized by hyperemic endometrial areas with central pallor, are found in up to 65% of women with confirmed CE [8].

CE has been associated with infertility, particularly in patients attempting to conceive naturally or in patients with recurrent in vitro fertilization (IVF) failure. Studies have shown that individuals with unexplained infertility have a higher incidence of CE compared to those without infertility, with one study reporting a diagnosis in 45% of individuals with recurrent implantation failure [9]. Additionally, studies have shown that individuals with non-*Lactobacillus*-dominated microbiota are associated with decreased rates of pregnancy in general [7]. The relationship between CE and infertility is still being explored, with several hypotheses offered to explain this link. One theory suggests that the chronic inflammation associated with CE disrupts endometrial regeneration, leading to the formation of adhesions and a hostile uterine environment that impairs fertility [9]. Another theory is that CE can alter the production of various cytokines which causes an abnormal leukocyte population within the endometrium as well as alter the secretion of various factors that are involved in endometrial receptivity [9]. These changes within the endometrium are hypothesized to cause failed implantation and thus infertility [9]. Although its association with infertility is not yet fully understood, CE treatment remains an important consideration in fertility care, and its concurrent presentation with bacteriospermia is clinically underreported.

## Case presentation

**Figure 1:**
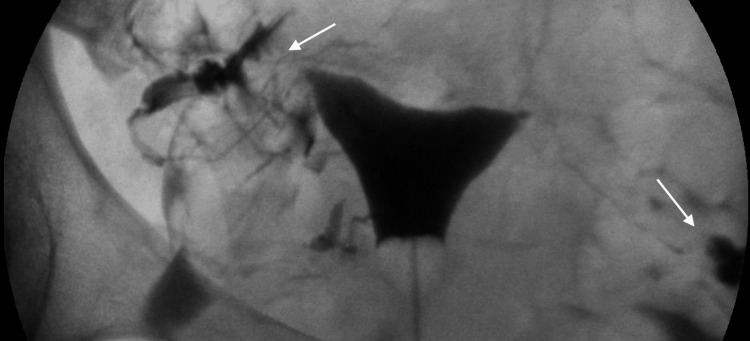
Normal HSG demonstrating tubal patency HSG of the patient demonstrates a uterus of normal size, shape, and contour. Fallopian tubes display a normal spill pattern bilaterally, which is indicated by the white arrows. HSG: hysterosalpingogram

A 35-year-old woman initially presented to the office for infertility evaluation after failing to conceive naturally with luteinizing hormone (LH) ovulation kits for 16 months. The patient's medical history was significant for two elective abortions and treatment for chlamydial infection. Her initial infertility workup included blood work to determine ovarian reserve, a hysterosalpingogram (HSG) to determine tubal patency, and a semen analysis on the patient's partner. The patient's blood work showed that she had an anti-Müllerian hormone (AMH) level of 4.8 ng/mL, indicating adequate ovarian reserve. The HSG demonstrated a uterus of normal size, shape, and contour and fallopian tubes with a normal spill pattern bilaterally (Figure 1). The patient's partner was uncircumcised and had a history of bacterial prostatitis. Semen analysis was normal with 82% motility (ref range: >42%), 121.6 million/mL concentration (ref range: >16 M/mL), and a total count of 182.5 million (ref range: >39 million). Based on these results, the couple was diagnosed with unexplained infertility and required further evaluation. 

The patient underwent genetic testing, which showed that she was a carrier for Alport's syndrome, an X-linked recessive disease. Based on the diagnosis of unexplained fertility and her carrier status, the patient was recommended to pursue fertility treatment with IVF following a pre-implantation genetic testing for monogenic disorders (PGT-M) analysis of the embryos. However, the patient and her partner opted to attempt ovulation induction with letrozole followed by intrauterine insemination (IUI) before considering IVF. Three attempts of letrozole with IUI were unsuccessful. 

In preparation for IVF, an EMB was performed during the follicular phase of the patient's cycle to determine the health of the uterus. The report from this biopsy reported a histological finding of fewer than five stromal plasma cells identified with CD138 staining per 10 HPF, which was a finding suggestive of CE. The histology photomicrographs of the EMB were not included in the report given to the provider. She was treated with azithromycin 250 mg once daily for two weeks due to previous intolerance to doxycycline, the recommended first-line treatment. The couple did not return for one year, at which point the patient underwent a second EMB, revealing five plasma cells with CD138 staining and again consistent with mild CE. She was treated again with azithromycin 250 mg once daily for two weeks. Due to the persistent nature of CE in the patient despite antibiotic therapy, a semen culture was performed on the partner's semen. The results from the semen culture showed a heavy growth of *E. faecalis* susceptible to penicillin and predictably ampicillin/sulbactam, amoxicillin/clavulanate, and piperacillin with a moderate growth of *Escherichia coli* susceptible to amoxicillin/clavulanate, cefazolin, and ciprofloxacin. The partner was treated with amoxicillin/clavulanic acid 875-125 mg twice daily for 10 days.

A third EMB and a second semen culture were done a year and a half after the initial presentation. The results of this EMB identified four stromal plasma cells with CD138 staining. Because this finding of fewer than five plasma cells is only suggestive of CE and considered negative, the patient was not treated, while her partner was being investigated as the source of infertility. Semen culture results still showed moderate growth of *E. faecalis* susceptible to penicillin and predictably ampicillin/sulbactam, amoxicillin/clavulanate, and piperacillin, although the *E. coli* was no longer present. The partner was subsequently treated with penicillin V potassium 500 mg once daily for 10 days. Repeat semen culture demonstrated moderate growth of *Staphylococcus* susceptible to ciprofloxacin and levofloxacin, treated with ciprofloxacin 500 mg taken twice a day for 10 days. At this point, repeat semen culture no longer detected the presence of any bacteria. Due to the persistent presence of bacteria in the semen, three vials of sperm were cryopreserved following the negative culture.

Two years after initial evaluation, the patient underwent a fourth EMB consistent with a diagnosis of CE and was treated with the same azithromycin regimen. Because of recurrent positive semen cultures and recurrent positive EMBs, the patient was advised to undergo an EMMA and ALICE testing in order to determine the precise bacteria that persisted despite antibiotic therapy. The first EMMA/ALICE test was done one week after ovulation and detected the presence of *E. coli* and *E. faecalis* in the uterus. These bacteria were the same bacteria present in her partner's semen cultures. The patient was subsequently treated with amoxicillin/clavulanic acid 875-125 mg twice a day for two weeks. The patient and her partner were advised to use condoms during intercourse to prevent the further spread of any bacteria from the partner's semen to the patient's uterus. 

The patient underwent a second EMMA/ALICE test with the detection of *E. faecalis* treated with moxifloxacin 400 mg taken once a day for two weeks. Upon request by the patient, her partner had a repeat semen culture, now demonstrating the presence of group B *Streptococcus*. The partner was treated with ampicillin 500 mg taken four times a day for three weeks. Subsequent EMMA/ALICE testing now detected the presence of *K. pneumoniae*. The patient was treated with amoxicillin/clavulanic acid 875-125 mg taken twice a day for two weeks, along with *Lactobacillus* supplements. Three years after the initial presentation, a final semen culture and fourth EMMA/ALICE test no longer detected the presence of any bacteria. The partner cryopreserved additional sperm at this time. 

During the course of CE treatment, the patient had undergone four egg retrieval processes. Of the retrieved eggs, the viable eggs were fertilized with the partner's cryopreserved sperm samples. Fertilized embryos underwent PGT-M analysis, yielding three unaffected male embryos, two unaffected female embryos, and four female carrier embryos. Two and a half years after the couple initially presented for infertility evaluation, a frozen embryo transfer with one of the unaffected male embryos was performed, and she became pregnant and delivered a healthy male. Table 1 summarizes the entire course of treatment including diagnostic evaluation with EMB, EMMA/ALICE, and semen culture, the organisms isolated and appropriate antibiotic therapy, and dates of egg retrieval, sperm cryopreservation, and embryo transfer.

**Table 1:** Chronological timeline detailing the clinical investigation with EMB, EMMA/ALICE, semen culture, antibiotic course, and IVF milestones IUI: intrauterine insemination; IVF: in vitro fertilization; CE: chronic endometritis; EMB: endometrial biopsy; EMMA: endometrial microbiome metagenomic analysis; ALICE: analysis of infectious chronic endometritis

Date	Diagnostic evaluation	Result	Treatment
11/2020	Initial fertility evaluation	Unexplained infertility	3 rounds of IUI with letrozole unsuccessful
5/2021	EMB #1 in preparation for IVF	Nondiagnostic for CE	Azithromycin
5/2022	Return to care		
	EMB #2	CE (5 plasma cells)	Azithromycin
	Semen culture #1	*E. coli* and *E. faecalis*	Amoxicillin/clavulanate
8/2022	EMB #3	Nondiagnostic of CE	
	Semen culture #2	E. faecalis	Penicillin
9/2022	Semen culture #3	Staphylococcus	Ciprofloxacin
11/2022	Semen culture #4	Negative	
	Sperm cryopreservation #1		
12/2022	EMB #4	CE (5 plasma cells)	
	EMMA/ALICE #1	*E. coli* and *E. faecalis*	Amoxicillin/clavulanate barrier protection
	Egg retrieval #1		
3/2023	EMMA/ALICE #2	E. faecalis	Moxifloxacin
	Semen culture #5	Streptococcus agalactiae	Ampicillin
	Egg retrieval #2		
5/2023	Egg retrieval #3		
6/2023	EMMA/ALICE #3	Klebsiella	Amoxicillin/clavulanate and *Lactobacillus* supplement
10/2023	Semen culture #6	Negative	
	EMMA/ALICE #4	Negative	
11/2023	Sperm cryopreservation #2		
	Egg retrieval #4		
	Embryos created		
1/2024	Healthy male embryo transferred		

## Discussion

Although CE is often discovered incidentally, this case demonstrates the importance of routine evaluation for CE with an EMB in a patient with unexplained infertility. The literature supports a higher prevalence of CE in individuals with infertility than in those without infertility [8]. In this case, the patient had recurrent failure to conceive with IUI despite patent fallopian tubes, adequate ovarian reserve, normal uterine anatomy, and a partner with sufficient semen analysis. Because initial testing ruled out major causes of infertility, further evaluation was warranted. During this continued investigation, the first EMB revealed fewer than five stromal plasma cells with CD138 staining, which was a finding suggestive of CE, although not diagnostic [6]. Empiric antibiotic therapy with azithromycin 250 mg once daily for two weeks was chosen to treat the suspected CE. Typically, standard treatment involves a 14-day regimen of doxycycline 100 mg twice daily [8-10]. Azithromycin was chosen in this patient case because it is better tolerated than doxycycline and has once-a-day dosing, increasing adherence to the medication regimen [11]. Metronidazole with either levofloxacin or ciprofloxacin can also be considered as second-line therapy [8-10]. In cases where these treatments fail, an EMMA/ALICE test may be conducted to identify the specific bacteria responsible and allow for targeted antibiotic therapy. However, it must be noted that although EMMA/ALICE testing can be useful in identifying the exact bacteria that could be contributing to the development of CE, there are limitations to this test as well. The bacteria that is present in the uterus is always evolving, and since the EMMA/ALICE test only captures the bacterial load in the uterus at a single point in time, it is possible that this test does not accurately determine the specific bacteria that may have caused the development of CE. 

After multiple EMBs consistently positive for CE and treatment with several courses of azithromycin, the patient underwent EMMA and ALICE testing to gain information for more targeted antibiotic therapy, which identified the presence of *E. coli* and *E. faecalis* in the uterus, the same bacteria present in her partner's initial semen cultures. There is not much published literature on the relationship between seminal microbiota and its correlation with CE. Our patient and her partner both tested positive for *E. coli* and *E. faecalis*; however, only our female patient presented with *K. pneumoniae*. 

The effect of bacteriospermia on sperm quality continues to be a highly debated topic. *Lactobacillus* has also been shown in semen cultures to be beneficial to sperm quality, whereas *U. urealyticum*, *M. hominis*, *Prevotella*, *E. coli*, and *E. faecalis* have negatively impacted sperm from mobility to concentration [12,13]. The male partner had a heavy growth of *E. faecalis* and a moderate growth of *E. coli*; however, he had a normal semen analysis. A 2022 publication detailing a retrospective single-center cohort study reported that asymptomatic bacteriospermia can lead to reduced sperm parameters. Particularly, when semen culture showed *E. faecalis*, there could be an association with decreased sperm concentration and low numbers of live spermatozoa on semen analysis [14]. However, our patient's partner had normal semen analysis and sperm parameters, and a semen culture was only obtained due to a CE that was unresolved after multiple rounds of antibiotic treatment. Our case suggests the possibility that asymptomatic bacteriospermia may contribute to the development of CE. 

Microbial dysbiosis likely induces the chronic inflammation of the uterine cavity seen with CE, a risk that is increased in those with a history of possible bacterial contamination of the uterus [3-5]. Bacteriospermia, particularly with culture demonstrating *E. faecalis*, has been associated with not only reduced sperm parameters but also reduced success of embryo transfer [14]. One study assessed coronal sulci bacteria in circumcised versus uncircumcised males to determine how male circumcision influences pathogen predominance in the penis. An association was reported between uncircumcised status and opportunistic growth of Gram-positive *S. aureus* and *Enterococcus* species, as well as Gram-negative *E. coli* and *Klebsiella* species in the coronal sulci [15]. Semen culture in this uncircumcised male partner with persistent bacteriospermia revealed these same pathogens, supporting the possible role of circumcision on the male genital tract and seminal fluid microbiota. While the effect of bacteriospermia on sperm quality remains under investigation, we propose a possible role of seminal microbiota in the development and persistence of CE in an asymptomatic couple with unexplained infertility. 

This case demonstrates an unusual occurrence of bacterial co-colonization of the endometrium and seminal fluid in a couple with unexplained infertility, suggesting a potential pathway for CE development from bacteriospermia. The patient's EMMA/ALICE tests and the partner's semen cultures revealed the presence of the same bacteria. While current literature does not identify the development of CE from the bacteria in a partner's semen, limited evidence supports an association between bacteria in semen and infertility [16]. In couples with unexplained infertility, thorough evaluation for CE with EMB and EMMA/ALICE can be performed in conjunction with a semen culture on the partner to explore this potential correlation. Patients with unexplained infertility can be evaluated for CE with an EMB, treated with first-line antibiotics once histologic CE is confirmed, and retested to ensure resolution. If CE does not resolve, then an EMMA/ALICE test can be considered to determine the presence of specific bacteria and guide antibiotic therapy. Persistent infection may warrant partner semen culture to guide further dual-partner treatment. 

Further research is necessary to uncover the relationship between bacteriospermia and the persistence of CE. Although the presence of specific seminal bacteria has been correlated with reduced sperm parameters and lower success rates of embryo transfer, these findings cannot be generalized to a broader infertility population. Several limitations should be acknowledged. In this case, quantitative microbial load data were not available which limits the interpretation of bacterial burden and its clinical relevance. The possibility of cervical or vaginal contamination of uterine samples cannot be excluded. Furthermore, it is difficult to ensure patient compliance with antibiotic therapy and abstinence periods or barrier contraception use to prevent re-infection. Larger, prospective studies incorporating standardized sampling methods, quantitative microbial analyses, and parallel evaluation of both partners' reproductive tract microbiomes are warranted to clarify the potential bidirectional relationship between bacteriospermia, CE, and fertility outcomes.

## Conclusions

We report an unusual case of bacterial co-colonization of the endometrium and seminal fluid in a couple with unexplained infertility. This case proposes the importance of treating CE in a patient with unexplained infertility. It is prudent to rule out any potential issues prior to conception with IVF, an expensive and arduous process. Patients can be evaluated for CE with an EMB, treated with first-line antibiotics, and retested to ensure resolution. If CE does not resolve, then an EMMA/ALICE test can be considered to determine the presence of specific bacteria and guide antibiotic therapy. A correlation was identified between the bacteria in the partner's semen and the bacteria responsible for the patient's CE, although this represents a single case study and this association has not been previously reported. Conducting a semen culture in conjunction with an EMB and EMMA/ALICE allows for the evaluation of bacterial co-colonization. If semen culture reflects the presence of bacteria, the partner can also complete a targeted antibiotic course, remain abstinent, or use condoms during intercourse to prevent further transmission or re-infection.
